# The Economic Burden of Alopecia Areata: Evidence from a Survey in Norway and Sweden

**DOI:** 10.2340/actadv.v106.adv-2025-0114

**Published:** 2026-02-16

**Authors:** Sofia Löfvendahl, Frida Hjalte, Ida Haggren, Evy-Ann Engdal Hamre, Flora Balieva, Marcus Schmitt-Egenolf

**Affiliations:** 1 The Swedish Institute for Health Economics, Lund, Sweden; 2 Division of Orthopaedics, Department of Clinical Sciences Lund, Lund University, Lund, Sweden; 3 Department of Economics, Lund University School of Economics and Management, Lund University, Lund, Sweden; 4 The Norwegian Alopecia Association, Oslo, Norway; 5 Department of Dermatology, Stavanger University Hospital, Stavanger, Norway; 6 Faculty of Health Sciences, Department of Public Health, University of Stavanger, Stavanger, Norway; 7 Centre for Pharmacoepidemiology, Karolinska Institutet, Stockholm, Sweden

**Keywords:** alopecia areata, resource use, societal costs, survey, Norway, Sweden

## Abstract

While international studies have assessed the economic burden of alopecia areata (AA), its societal costs have not been quantified in a Nordic context. We conducted a cross-sectional survey among adults with self-reported AA in Norway and Sweden, recruited via a patient organization and social media. A total of 329 respondents (263 from Norway, 66 from Sweden) provided information on demographics, disease characteristics, healthcare utilization, out-of-pocket expenses, productivity losses and treatment satisfaction. Costs were estimated from a societal perspective, combining direct medical, direct non-medical and indirect costs from reduced productivity. The annual mean total cost of AA was €7,677 in Norway and €12,582 in Sweden, with indirect costs (61–64% of the total) as the largest component, primarily driven by presenteeism and long-term sick leave. A notable finding is the significant out-of-pocket costs. In Norway, individuals paid about 65% of direct costs themselves, in Sweden about 50%. Dissatisfaction with treatment and healthcare support was widespread. Only a minority received systemic therapies, and treatment frequency with Janus kinase inhibitors was low, likely due to lack of reimbursement. AA imposes a considerable societal and individual economic burden in Norway and Sweden, underscoring the need for better therapies, healthcare support and policy recognition of its impact.

SIGNIFICANCE

This study shows that alopecia areata (AA) places a significant economic burden on society, as well as both economic and personal burdens on individuals in Norway and Sweden. Many people with AA experience reduced work ability and spend considerable amounts of money on wigs and cosmetics while feeling dissatisfied with current treatments and healthcare support. Although some therapies are available and reimbursed, few individuals seem to receive effective medical treatment, highlighting the need for improved care, better access to effective options and policies that fully recognize the impact of living with AA.

Alopecia areata (AA) is an autoimmune disorder mediated by T-lymphocytes, causing hair follicles to prematurely enter a resting phase, leading to non-scarring hair loss (1). The severity of hair loss ranges from small scalp patches AA and diffuse AA to more extensive forms, including AA totalis (entire scalp) and AA universalis (all scalp and body hair) (1). Global prevalence estimates range from 0.02% to 0.21% (2), with an annual incidence of 20.9 per 100,000 person-years and a lifetime risk of 2.1% (3). Early onset increases the risk of extensive disease (2, 4).

The course of AA is unpredictable, often involving cycles of regrowth and relapse (1). Although the aetiology remains unclear, factors such as emotional stress, pregnancy and infections may increase susceptibility (5).

While not life-threatening, AA significantly impairs health-related quality of life (HRQoL), particularly due to stigmatization and mental health issues (6–9). Severe, prolonged or recurrent disease and comorbidities further worsen HRQoL (6).

Treatments include topical, intralesional and systemic corticosteroids, topical immunotherapy, minoxidil and methotrexate; however, there remains a substantial unmet therapeutic need due to limited efficacy and access (10). Recently, several promising Janus kinase (JAK) inhibitors have emerged, but their use remains restricted (10, 11). JAK-inhibitors are currently excluded from the pharmaceutical reimbursement scheme both in Sweden (12, 13) and Norway (14, 15) due to insufficient documented evidence of cost-effectiveness. In the absence of effective treatments, many individuals turn to solutions, such as wigs and cosmesis, which are often associated with high out-of-pocket costs (16).

Several studies have examined AA-related resource use and costs (16–22), focusing mainly on healthcare resource use (17, 20, 21) and out-of-pocket expenses (7, 16, 19, 22, 23), with fewer addressing lost productivity (17, 18, 24). The societal economic burden of AA has not been examined within the Nordic context. This survey-based study, therefore, aimed to estimate the societal economic burden of AA in Norway and Sweden and to assess how it varies by background and disease-related characteristics.

## METHODS

### Study design

This cross-sectional online survey targeted individuals in Norway and Sweden with self-reported AA. In Norway, the Norwegian Alopecia Association (Alopeciaforeningen) distributed a link to the survey webpage to all members with a registered email address – 812 out of a total of 849 members. In Sweden, where no formal patient organization exists, the survey was promoted in the closed Facebook group “Alopecia Sverige-community” (≈3,000 members). Two reminders were sent/published in both countries. Inclusion criteria were age ≥18 years and self-reported AA, without requiring a formal medical diagnosis. Participation was voluntary and uncompensated. Data was collected from May to July 2024.

### Survey questionnaire

The web-based questionnaire collected information on background (age, sex, education, employment status), AA disease course (diagnosis, duration), and resource use associated with AA, including healthcare use, out-of-pocket expenses and productivity loss. Formal healthcare utilization (visits, medication and aids such as wigs) and out-of-pocket expenses (e.g. hair appointments, headwear, cosmesis) were assessed for the preceding 12 months. Satisfaction with AA treatment and with the healthcare system’s attitude was measured on a Likert scale (1=very dissatisfied to 5=very satisfied).

### Cost calculations

Costs were estimated by multiplying reported resource use and salary data with unit prices from regional and national price lists and pharmaceutical retail data (Tables SI and SII), (25–34). Productivity loss was calculated based on reported employment status and workdays missed due to AA. Productivity loss encompassed both short-term absenteeism, including sick pay provided by the employer and/or sickness benefit from the social insurance system, and long-term absenteeism, including sickness and activity compensation from the social insurance system. It also included presenteeism, defined as reduced productivity while at work. All measures reflected the impact of AA over the previous 12 months. Productivity loss was valued using the sex- and age-adjusted average wage (35, 36) plus payroll taxes (37, 38) applying the human capital approach (39). Presenteeism was valued similarly, weighted by the Likert score (0–10) for reduced productivity. Salaries were adjusted to 2024 levels. All costs were converted to euros using the average 2024 exchange rates: 1EUR=11.6290 NOK and 11.4325 SEK (40).

### Statistical analysis

We described the study populations, including demographics, healthcare use, medication, out-of-pocket expenses, productivity losses and associated costs. Both resource use and costs were presented as mean annual values per respondent. We also applied multiple regression models to examine the associations between population characteristics and total costs.

Descriptive statistics (mean with standard deviation (SD), median with interquartile range (IQR), min–max) are reported. Semi-logarithmic linear regression (y=ln(cost)) was used to assess associations between total costs and sex, age (four categories, 40–59 years as reference), disease duration (quartiles; 0–5 years as reference), education (four levels; “high” as reference), alopecia type (0=totalis/universalis, 1=only AA), and treatment satisfaction (three levels; “neither satisfied nor dissatisfied” as reference). Individuals with missing values were excluded. The Norwegian and Swedish populations were analysed and presented separately, as comparing the 2 countries was not an objective of the study. *p*-values are reported for the regression analyses conducted within each country to quantify the strength of the associations and to provide an indication of statistical uncertainty. Analyses were performed using Stata/IC 14.2 (StataCorp, College Station, TX, USA).

### Ethical approvals

The study was approved by the Swedish Ethical Review Authority (Ref. 2023-05941-01). In Norway, the Regional Committees for Medical and Health Research Ethics deemed the project outside the scope of medical and health research and not subject to formal approval (Ref. No 670096). Informed consent was obtained from all participants before participation.

## RESULTS

### Characteristics of the study populations

A total of 479 individuals accessed the questionnaire; 150 were excluded due to ineligibility or incomplete responses. The final study population comprised 329 respondents: 263 from Norway and 66 from Sweden (Fig. S1). Most were women (>93%), with a mean (SD) age of 50.3 (15.3) years in Norway and 43.0 (12.8) in Sweden (Table I). The majority had >12 years of education and were employed. AA duration exceeded 15 years in 48% of Norwegians and 42% of Swedes. Nearly all reported physician-diagnosed and active AA. Multiple subtypes were reported by just over 10% in Norway and nearly 30% in Sweden.

**Table I.** Characteristics of the study populations

	Norway (*n*=263)	Sweden (*n*=66)
Female, *n* (%)	245 (93.2)	63 (95.5)
Age, years, mean (SD)	50.3 (15.7)	43.0 (12.8)
Age group, *n* (%)		
18–39 years	73 (28.0)	26 (39.0)
40–59 years	104 (40.0)	33 (50.0)
≥60 years	86 (33.0)	7 (11.0)
Level of education, *n* (%)		
Low (≤9 years)	13 (4.9)	3 (4.5)
Medium (10–12 years)	73 (27.8)	24 (36.4)
High (>12 years)	167 (63.5)	36 (54.5)
Other	6 (2.3)	3 (4.5)
Missing	4 (1.5)	0 (0.0)
Occupation, *n* (%)		
Employed/self-employed	183 (69.6)	52 (78.8)
Student	10 (3.8)	4 (6.1)
Retired	42 (16.0)	2 (3.0)
Sickness or activity compensation^a^	24 (9.1)	7 (10.6)
Unemployed	1 (0.4)	1 (1.5)
Other	3 (1.1)	0 (0.0)
Time since debut of symptoms, *n* (%)		
<1 year	6 (2.3)	4 (6.1)
1–5 years	49 (18.6)	18 (27.3)
6–10 years	35 (13.3)	11 (16.7)
11–15 years	50 (19.0)	6 (9.0)
>15 years	123 (46.8)	27 (40.9)
Physician-diagnosed AA, *n*, (% yes)	255 (97.0)	66 (100.0)
Years since diagnosis, mean (SD)	15.8 (10.8)	14.0 (11.1)
Years since diagnosis, *n* (%)^b^		
0–5 years	64 (25.8)	22 (33.9)
6–14 years	65 (26.2)	16 (24.6)
15–28 years	54 (21.8)	14 (21.5)
> 29 years	65 (26.2)	13 (20.0)
Missing, *n*	15	1
Currently active alopecia areata, *n* (%)	240 (91.3)	64 (97.0)
Diagnosis type, *n* (%)^c^		
>1 diagnosis reported	35 (13.7)	18 (27.2)
Alopecia areata	130 (49.4)	47 (71.2)
Alopecia totalis	47 (17.9)	12 (18.2)
Alopecia universalis	126 (47.9)	31 (47.0)
Missing, *n*	8	0

^a^In Sweden: sjuk- och aktivitetsersättning, in Norway: uføretrygd eller arbeidsavklaringspenger. ^b^Quartile strata. ^c^Multiple answers allowed.

SD:standard deviation.

### Resource use – healthcare visits and drug use

In Norway, 46% reported healthcare visits in the past 12 months, mainly to physicians (43%), with a mean (SD) of 5.9 (7.1) visits among those seeking care (Table II). In Sweden, 68% reported visits, primarily to physicians (64%), with a mean (SD) of 4.4 (7.3) visits. Consultations with other health professionals were more frequent in Sweden (23%) than in Norway (11%).

**Table II.** Healthcare visits and medication use due to alopecia areata in the past 12 months

	Norway, *n*=263		Sweden, *n*=66	
Healthcare visits	Number (%) of respondents with visits	Mean (SD) number of visits among respondents with visits	Number (%) of respondents with visits	Mean (SD) number of visits among respondents with visits
*Any healthcare visits*	120 (45.6)	8.3 (10.8)	45 (68.2)	7.9 (14.0)
Physician visits^a^	114 (43.4)	5.9 (7.1)	42 (63.6)	4.4 (7.3)
Nurse visits	14 (5.3)	3.5 (3.0)	6 (9.1)	1.5 (0.5)
Other healthcare professional^b^	29 (11.0)	9.5 (10.0)	15 (22.7)	10.5 (13.2)
Type of medication use	Number (%) of respondents with use	Mean (SD) number of months with use among respondents with any use	Number (%) of respondents with use	Mean (SD) number of months with use among respondents with any use
*Any medication use*	59 (22.4)	NA	16 (24.2)	NA
Topical steroids	35 (13.3)	6.13 (4.40)	14 (21.2)	6.4 (4.1)
Oral steroids	11 (4.2)	5.73 (4.14)	0 (0)	NA
Systemic treatments	23 (8.7)	8.2 (4.5)^c^	4 (6.0)	6.5 (7.8)^c^

^a^Physician visits=dermatologist, primary care physician, psychiatrist, or another specialised physician. ^b^Other healthcare professional, e.g. psychologist, physiotherapist, social worker, nutritionist. ^c^Excluding JAK-inhibitors.

NA:not applicable.

Medication use was reported by 22% in Norway and 24% in Sweden, most often topical steroids. Although few respondents reported use of systemic medications (23 in Norway and 4 in Sweden), 8 individuals (35%) in Norway and 2 (50%) in Sweden among them reported using JAK-inhibitor. A small number of respondents reported corticosteroid injections, phototherapy or laser therapy, but the frequency of use was low. Complementary use of vitamins, herbal products or minoxidil was reported by nearly one-third in Norway and two-thirds in Sweden.

Most used some form of headgear (85% Norway, 88% Sweden) (Table III). Wigs were predominant, followed by hats, shawls or postiches; toupees were rare. Hair products and cosmesis were also commonly used, particularly in Sweden. About one-third of Norwegians and one-quarter of Swedes reported extra hairdresser visits, while 40% and 35%, respectively, reported camouflage tattoos; no respondents had undergone hair transplantation.

**Table III.** Resource use for hair loss camouflage due to alopecia areata in the past 12 months

	Norway, *n*=263	Sweden, *n*=66
Headgears		
*Any use of headgears, n (%*)	221 (84.7%)	57 (86.4%)
Postiches, *n* (%)	21 (8.0%)	7 (10.6%)
Wigs, *n* (%)	195 (74.1%)	44 (66.7%)
Toupées *n* (%)	1 (0.4%)	3 (4.5%)
Other headgears (mostly hats, caps, shawls), *n* (%)	53 (19.8%)	17 (25.8%)
Hair loss camouflage products or services		
Any hair loss camouflage*, n (%*)	208 (79.1%)	64 (97.0%)
Hair products, *n* (%)	113 (43.0%)	45 (68.2%)
Cosmesis, *n* (%)	118 (44.9%)	34 (51.5%)
Extra visits to hairdresser, *n* (%)	83 (31.6%)	16 (24.2%)
Tattoos, *n* (%)	105 (39.9%)	23 (34.9%)
Other (minoxidil, vitamins, herbal medicines), *n* (%)	81 (30.8%)	38 (57.6%)

### Satisfaction with treatment and attitude from the healthcare system

Most respondents were dissatisfied with their AA treatment, 84% in Norway and 89% in Sweden rated it at the lower end of the Likert scale (1, 2) ([Supplementary-material SP1] [Supplementary-material SP1] Figs S2 and S3). Similarly, dissatisfaction with the overall healthcare system was common (84% Norway, 74% Sweden), while just over 10% of Norwegians and 16% of Swedes were neutral, and 5% and 9% reported some satisfaction.

### Productivity loss

Most respondents were employed (70% in Norway and 79% in Sweden) (Table IV). Fewer than 10% of employed respondents reported receiving sickness benefit or sickness and activity compensation. Short-term sick leave, covered by sick pay, was reported by 12% of employed Norwegians and 10% of Swedes, with a mean duration of 11 and 14 days, respectively.

**Table IV.** Productivity loss due to alopecia areata in the past 12 months

	Norway, *n*=263	Sweden, *n*=66
Employed or self-employed *n* (%)	183 (69.6)	52 (78.8)
Full-time *n* (%)	148 (80.9)	43 (82.7)
Part-time *n* (%)	35 (19.1)	9 (17.3)
Reported productivity loss among those employed	*n*=183	*n*=52
Sickness benefit^a^, *n* (%)	5 (2.7)	4 (7.7)
Sickness and activity compensation^b^ *n* (%)	5 (2.7)	1 (1.9)
Sick pay^c^ due to AA, *n* (%)	22 (12.0)	5 (9.6)
*Number of days*		
Mean (SD)	10.8 (24.8)	14.4 (18.0)
Median (Q1; Q3)	4.5 (3, 7)	10.0 (4, 10)
Min; max	1; 120	2; 46
Presenteeism due to AA, *n* (%)	143 (78.1)	37 (71.2)
*Number of days*		
Mean (SD)	60.6 (57.4)	65.9 (77.5)
Median (Q1; Q3)	51 (40.8; 51)	51 (25; 51)
Min; max	1.8; 251	2.8; 251
*Effect on productivity (VAS 0–10, 10=completely unable*)		
Mean (SD)	2.7 (2.4)	3.8 (2.7)
Median (Q1; Q3)	2 (0;5)	4 (2, 5)
Min; max	0; 9	0; 10

^a^short-term absenteeism compensated by the social insurance system. ^b^long-term absenteeism compensated by the social insurance system. ^c^short-term absenteeism compensated by the employer.

AA:alopecia areata; Q1:percentile 1; Q3:percentile 3; SD:standard deviation.

Presenteeism was common, reported by 78% of employed Norwegians and 71% of Swedes (Table IV). They worked a mean of 61 and 66 days with AA-related difficulties, with mean productivity impacts of 2.7 and 3.8, respectively. Notably, one-third of Norwegians and one-tenth of Swedes reported no productivity impact. AA also affected usual daily activities outside of work, reported by 80% in Norway and 85% in Sweden. On a 0–10 scale Likert scale, mean (SD) impact was 3.5 (2.8) in Norway and 4.9 (3.0) in Sweden (data not shown).

### Costs

In Norway, the mean annual total cost per respondent in the total sample was €7,677 (SD 11,824), of which 39% (€2,975, SD 3,288) were direct costs and 61% (€4,701, SD 10,730) were indirect costs (Table V).

**Table V.** Descriptive statistics for cost components for individuals with alopecia areata in Norway and Sweden (in € per year)

Cost components	Norway, *n*=263			Sweden, *n*=66				
	Mean (SD), €	Median (Q1; Q3), €	No. with any use/productivity loss	Mean (SD), €	Median (Q1; Q3), €	No. with any use/productivity loss	
Direct medical cost							
Healthcare	310(773)	0 (0; 278)	120/263 (7.6%)	1,907(4,727)	525(0; 1,574)	44/66 (66.7%)	
Medications^a^	78(552)	0 (0; 0)	53/263(20.2%)	196 (1,255)	0 (0; 0)	15/66 (22.7%)	
Headgear	1,942(2,651)	1,685(539; 2,580)	221/263 (84%)	1,616 (1,493)	1,487(472; 2,344)	57/66 (86.4%)	
Hair loss camouflage products or services^b^	645(831)	430 (42; 860)	208/263 (79.1%)	766(933)	424 (174; 875)	64/66 (97%)	
Indirect cost							
Short term sick leave	289(2,376)	0 (0; 0)	22/183 (12%)	273(1,502)	0(0; 0)	5/52 (9.6%)	
Long term sick leave	1,121(6,837)	0(0; 0)	9/183(4.9%)	3,423(13,325)	0(0; 0)	5/52 (9.6%)	
Presenteeism	3,291(8,070)	0(0; 3,553)	99/183 (54.1%)	4,401(10,194)	0(0; 0)	32/52 (61.5%)	
Total annual cost	7,677(11,824)	3,381(1,308; 8,373)	263	12,582 (17,241)	5,335 (2,361; 13,845)	66	

^a^In Norway, five individuals reported receiving corticosteroid injections; these costs are not included in calculations. One individual reported using JAK-inhibitor without reporting any associated costs. In Sweden, one individual reported corticosteroid injections; the costs of these are not included. ^b^Minoxidil, vitamins, herbal medicines, hair products, cosmesis, extra visit to hairdresser, tattoos. Five individuals in Norway and one individual in Sweden who reported resource use did not report any costs. ^c^Sickness benefit and sickness and activity compensation.

AA:alopecia areata; SD:standard deviation.

When the analysis of indirect costs was restricted to only employed (70% in Norway), the mean indirect cost rose to €6,757 (SD 12,321). In Sweden, the mean annual total cost per respondent was €12,582 (SD 17,241), with direct costs of €4,485 (SD 5,216, 36%) and indirect costs of €8,096 (SD 16,426, 64%). Restricted to employed (79%), the mean indirect cost increased to €10,276 (SD 17,918). There was considerable variation in individual costs. Median (IQR) total costs were €3,381 (1,308–8,373) in Norway and €5,335 (2,361–13,845) in Sweden.

In Norway, individuals paid 65% of direct costs out-of-pocket (€1,928, SD 2,884), while in Sweden the share was approximately 50% (€2,227, SD 2,351) (data not shown). Out-of-pocket costs included costs for hair loss camouflage products or services and part of headgear costs, with partial reimbursement for wigs in both countries.

**Fig. 1.**
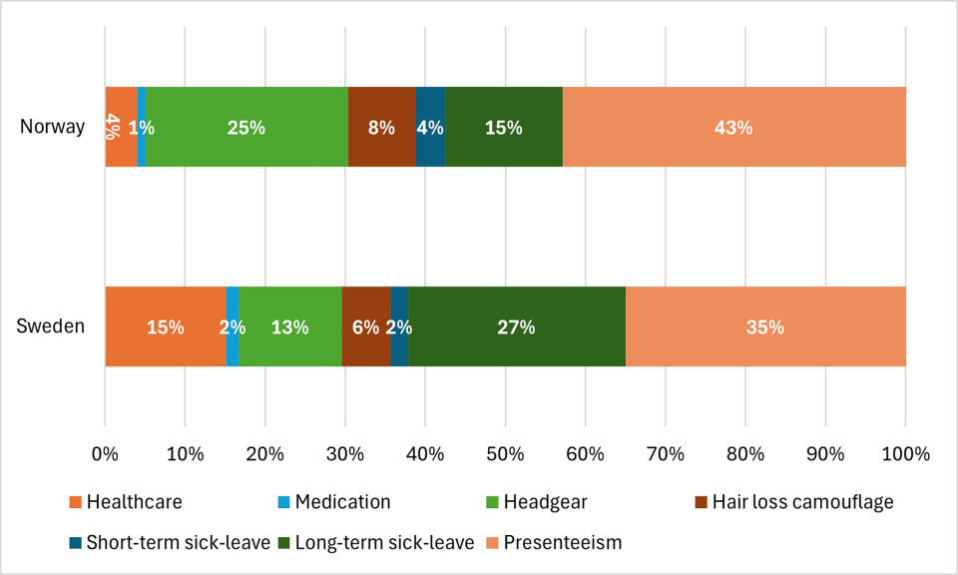
Components of annual costs for alopecia areata for defined study populations in Norway and Sweden, respectively.

In Norway, the largest cost component was presenteeism (43%), followed by headgear (25%) and long-term sick leave (15%), with smaller shares for other cost components (Fig. 1). In Sweden, long-term sick leave (27%) and presenteeism (35%) were the largest components, followed by healthcare (15%).

For Norway, regression analysis showed that younger age (18–39 vs 40–59) and both dissatisfaction and satisfaction with treatment (vs neutral) were associated with higher costs (Table SIII) whereas male sex and age 60–89 (vs 40–59) were associated with lower total costs. In Sweden, only dissatisfaction was associated with higher costs Table SIV.

## DISCUSSION

This study quantifies the societal burden of AA in a Nordic context, capturing direct and indirect costs. Findings highlight AA-related costs in Norway and Sweden, largely driven by reduced work productivity and out-of-pocket spending on wigs and camouflage products, underscoring both the economic burden and unmet care needs and support for affected individuals.

The annual total costs of AA averaged €7,677 per respondent in Norway and €12,582 in Sweden, with most costs from indirect sources and direct costs accounting for about one-third of the economic burden. Among cost components, presenteeism was the largest in Norway (43%), whereas in Sweden both long-term sick leave (27%) and presenteeism (35%) were the main contributors. These findings align with studies from the United States and Japan, which also found indirect cost to be the primary driver of the AA burden (17, 18, 24). For example, Gandhi et al. (18) reported that in the US, emotional distress and stigmatization significantly reduced workplace productivity.

The high prevalence of presenteeism highlights the psychological and social impact of AA at work. While the daily effect on productivity was moderate, its cumulative impact was substantial. These findings reinforce that, although not life-threatening, AA can severely impair HRQoL and cause psychological distress (6–8). Emotional stress, embarrassment and social withdrawal are likely to reduce engagement and efficiency. Beyond work, AA also affected leisure and daily activities, with many reporting greater impact outside work, underscoring its pervasive psychosocial consequences (7).

A notable finding is the significant out-of-pocket costs, particularly for headgear and camouflage products. In Norway, individuals paid about 65% of direct costs themselves, and in Sweden, about 50%, reflecting limited public reimbursement (8, 16). Both countries partly reimburse wigs, but most other expenses are self-funded. Similar patterns have been reported in Finland, Germany and the US, where individuals with AA spend substantial amounts on wigs, cosmesis products and other coping strategies (7, 16, 19, 22).

In a Finnish study based on data from 2023 (7) – an online survey of 226 respondents with AA (all subtypes), recruited through a patient organization and social media – participants reported spending an average of more than €600 out-of-pocket in the preceding year on wigs and an additional €800 on permanent pigmentation. These amounts were slightly lower than the out-of-pocket expenses for similar measures reported by Norwegian (≈ €2,600) and Swedish (≈ €2,300) respondents in the present study. These findings suggest a need for policymakers to consider more comprehensive support for individual appearance needs as part of standard care.

Healthcare utilization rates were relatively low: less than half of respondents in Norway and about two-thirds in Sweden reported AA-related healthcare visits in the preceding year, mainly to physicians. For Norway, these findings are consistent with a recent social media survey conducted in Norway and Denmark, which reported that only 36% of respondents had any healthcare contact within the past 12 months. Among those with visits, the median numbers were 2 to dermatologists, 2 to general practitioners and 4.5 to nurses. These figures closely correspond to the present Norwegian population, which observed median values of 2, 3 and 3.5 visits, respectively (data not shown) (9).

Only a minority in our study reported systemic therapies, highlighting the gap between clinical needs and available treatments. The limited use of systemic therapies, such as corticosteroids, methotrexate or JAK-inhibitors, aligns with concerns about their efficacy, tolerability and accessibility (10, 11). The very low prescription of JAK-inhibitors reflects their high cost, lack of reimbursement and uncertainty about long-term benefits (10, 11). Importantly, the high levels of dissatisfaction with medical treatment may stem partly from these therapeutic limitations, underscoring the need for better, more sustainable treatment strategies. These findings echo earlier reports that individuals often perceive AA treatment as ineffective and experience stigmatization or lack of understanding from the healthcare system (8, 10). This dissatisfaction may contribute to the observed low uptake of medical therapies and high reliance on camouflage and other coping strategies.

Differences between Norway and Sweden warrant consideration. Swedish respondents incurred higher direct and indirect costs, driven by greater resource use and higher unit prices for direct expenses. Although productivity loss unit prices were higher in Norway, total indirect costs were greater in Sweden, reflecting a larger proportion of respondents reporting some form of productivity loss.

Additionally, the differences may reflect variations in work culture, healthcare access, or the younger age profile of the Swedish sample. Younger individuals may be more likely to remain in the workforce and experience presenteeism or take formal sick leave, which would inflate indirect costs compared to an older cohort. Furthermore, cultural factors or differences in reimbursement schemes may influence how individuals manage their condition and engage with their healthcare services.

Regression analyses identified younger age and both dissatisfaction and satisfaction with treatment (vs neutrality) as being associated with higher costs in Norway. In Sweden, only dissatisfaction was associated with higher costs. Dissatisfaction may reflect greater disease activity or unmet needs, leading to higher resource use, absenteeism and presenteeism. Interestingly, satisfied respondents in Norway also had higher costs, possibly because those who accessed more and better treatments incurred greater expenses.

This study has several strengths. In Norway and Sweden, it captures both direct and indirect costs from a societal perspective, providing a comprehensive estimate of the economic burden. It is one of the few European studies on this topic, filling an important literature gap. The use of validated cost-estimation methods and country-specific unit costs and wages enhances credibility. High response rates in Norway, likely facilitated by distributing the survey through the Norwegian Alopecia Association, are another strength.

However, limitations exist. The cross-sectional, self-reported design may introduce recall bias. Recruitment via patient organizations and social media may select for more engaged or severely affected individuals, potentially overestimating costs. The high proportion of women and long disease duration in Norway suggest limited generalizability. Lack of clinical verification restricted analysis by subtype and severity, and the small Swedish sample limited precision. Finally, the study focused on resource use and costs without assessing HRQoL, which would provide a more comprehensive evaluation of the individual burden beyond economic impacts.

Despite these limitations, our findings have important implications. The high economic burden, dominated by indirect costs and out-of-pocket spending, highlights the unmet needs of AA populations in the Nordic setting. Policymakers and healthcare providers should consider strategies to reduce this burden, such as improving access to effective medical treatments, increasing reimbursement for other solutions and providing better psychological and occupational support. These findings may also inform cost-effectiveness analyses of emerging therapies, such as JAK-inhibitors, which have shown promise in moderate-to-severe AA but remain excluded from reimbursement due to insufficient documented cost-effectiveness. Our results suggest that more effective therapies could help offset costs by reducing productivity losses and out-of-pocket expenditures.

In conclusion, this study demonstrates that AA imposes a sizable economic burden in 2 designated populations in Norway and Sweden, mainly through reduced productivity and out-of-pocket spending on non-medical solutions. These findings underline the need for better access to effective treatments, stronger healthcare support and policies that reflect the full impact of AA. Future research should aim to validate these findings in larger, more representative populations and further examine the societal impact of emerging therapies incorporating patient-reported outcomes alongside cost data.

## Data Availability

The data that support the findings of this study are available from the corresponding author upon reasonable request.
