# Supplementary material for: The Economic Burden of Alopecia Areata: Evidence from a Survey in Norway and Sweden
Source: Acta Derm Venereol. 2026 Feb 16;106:0114. doi: 10.2340/actadv.v106.adv-2025-0114 (PMC12909710; doi:10.2340/actadv.v106.adv-2025-0114)
Supplement: Supplementary file 1. [file ActaDv-106-0114-s0001.docx]

**E-supplements:** 1 supplement word file (with several Figures and Tables)

# SUPPLEMENTAL FIGURE LEGENDS

## Fig. S1. Study population.


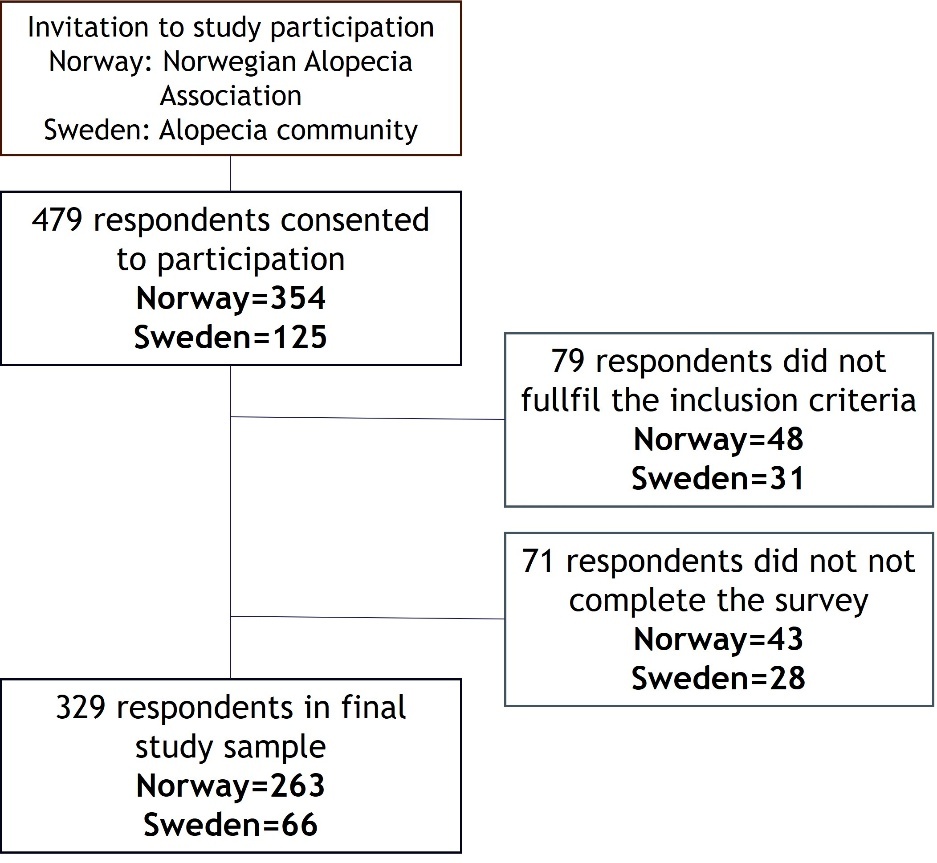


## Fig. S2. Satisfaction with treatment and attitude from the healthcare system, Norway.

AA=Alopecia areata

## Fig. S3. Satisfaction with treatment and attitude from the healthcare system, Sweden.

AA=Alopecia areata

# SUPPLEMENTAL TABLE LEGENDS

## Table SI. Unit costs. Price collection in October 2024 to February 2025.

Abbreviations: SEK=Swedish kronor, NOK=Norwegian kronor, SE=Sweden, NO=Norway

| **Resource use** | **Unit** | **Cost SE (SEK)** | **Reference SE** | **Cost NO (NOK)** | **Reference NO*** |
| --- | --- | --- | --- | --- | --- |
| **Healthcare visits** | | | | | |
| Dermatologist | Per visit | 6,000 | (31) | 1,321 | (32)  2ad + 4a1 + 3bd |
| Psychiatrist | Per visit | 6,864 | (31) | 1,321 | (32)  2ad + 4a1 + 3bd |
| Another specialist physician | Per (first) visit | 6,230 | (31) | 1,321 | (32)  2ad + 4a1 + 3bd |
|  | Per (return) visit | 3,542 | (31) |  |  |
| General practitioner | Per visit | 1,987 | (31) | 595 | (32)  2ad + 2cd |
| Nurse | Per visit | 819 | (31) | 475 | (33)  S2a +S2c |
| Specialist nurse | Per visit | 1,426 | (31) | 475 | As nurse |
| Psychologist | Per visit | 4,661 | (31) | 1,200 | (34)  22a |
| Social worker | Per visit | 1,426 | (31) | 475 | As nurse |
| Dietician | Per visit | 1,426 | (31) | 475 | As nurse |
| **Treatments** | | | | | |
| Phototherapy | Per treatment | 1,541 | (35) | 1,541 | As in Sweden |
| Laser treatment | Per treatment | 7,032 | (31) | 7,032 | As in Sweden |
| **Medication** | | | | | |
| Topical steroids | Per 100 g =  per 6 months for AA, per 2 months for AT and AU | 104 | betametason, cream, 0.1%, 100 g  (36) | 123 | hydrocortisone butyrate, Liniment opplösning, 0.1%, 100g  (37) |
| Oral steroids | Per 100 tablets =  per 3 months (SE) = per 2 months (NO) | 174 | prednisolone, tablet, 10 mg, 100 tablets  (36) | 136 | prednisolone, tablet, 5 mg, 100 tablets  (37) |
| Systemic treatments | Per 100 tablets =  per 4 months | 151 | methotrexate, tablet, 2.5 mg, 100 tablets  (36) | 336 | methotrexate, tablet, 2.5 mg, 100 tablets  (37) |
| **Hair replacements** | | | | | |
| Public cost  (age >= 30 years) | Per person who received public funds to acquire hair replacement | 7,500 | (38) | 6,265 | (39) |
| Public cost  (age < 30 years) |  |  |  | 25,000 |  |

* All costs according to the “normaltariffen” have been multiplied by 2 in accordance with the guidelines (retningslinjer). All time supplements in the standard tariff and Lovdata (3bd, 2cd, 4a1, S2a, S2c, 22a) have been multiplied by 0.41 because 41% of all visits to general practitioners (fastlege) include a time supplement (40)

## Table SII. Salary data

| **Age** | **Male** | **Female** |
| --- | --- | --- |
| **Price per day, Sweden (SEK)*** | | |
| 18-24 years | 2,164 | 2,049 |
| 25-34 years | 2,712 | 2,529 |
| 35-44 years | 3,184 | 2,857 |
| 45-54 years | 3,489 | 3,025 |
| 55-64 years | 3,360 | 2,933 |
| 65-66 years | 3,093 | 2,925 |
| **Price per day, Norway (SEK)**** | | |
| 0-24 years | 2,581 | 2,407 |
| 25-29 years | 3,441 | 3,274 |
| 30-34 years | 3,843 | 3,599 |
| 35-39 years | 4,163 | 3,800 |
| 40-44 years | 4,419 | 3,976 |
| 45-49 years | 4,620 | 4,076 |
| 50-54 years | 4,744 | 4,132 |
| 55-59 years | 4,734 | 4,126 |
| 60+ | 4,710 | 4,051 |
| * Calculate from mean monthly salary before tax 2022 ([SCB](https://www.statistikdatabasen.scb.se/pxweb/sv/ssd/START__AM__AM0110__AM0110A/LonYrkeAlder4A/table/tableViewLayout1/)), Price deflator = Consumer price index (KPI) July 2024 / KPI July 2022 = 1.121 ([SCB](https://www.scb.se/hitta-statistik/statistik-efter-amne/priser-och-konsumtion/konsumentprisindex/konsumentprisindex-kpi/pong/tabell-och-diagram/konsumentprisindex-kpi/kpi-faststallda-tal-1980100/)), Mean social tariffs = 1.421 ([Ekonomifakta](https://www.ekonomifakta.se/sakomraden/skatt/skatt-pa-arbete/sociala-avgifter-over-tid_1209267.html), [SCB](https://www.scb.se/contentassets/b49d7efc2653457f8179f18461d2bf38/am0208_2018a01_sm_am33sm2001.pdf)), Yearly working days: 251  ** Calculated from mean monthly salary before tax 2023 ([SSB](https://www.ssb.no/en/statbank/table/11421/tableViewLayout1/)), Price deflator = KPI July 2024 / KPI July 2023 = 1.028 ([SSB](https://www.ssb.no/kalkulatorer/priskalkulator)), Social tariffs Norge: 1.4 (28), Yearly working days: 251 | | |

## Table SIII. Linear regression of factors influencing total cost of alopecia areata, Norway (cost is in logarithm form)

| **Variables** | **β** | **p-value** | **95% CI** |
| --- | --- | --- | --- |
|  |  |  |  |
| **Male (vs female)** | -4.293 | **<0.001** | -5.186 - -3.401 |
| **Age (years)** |  |  |  |
| 18-39 | 0.645 | **0.018** | 0.111 - 1.180 |
| 40-59 (reference) |  |  |  |
| 60- | -1.172 | **<0.001** | -1.689 - -0.655 |
| **Years since diagnosis** |  |  |  |
| Quartile 1 (0-5 years) (reference) |  |  |  |
| Quartile 2 (6-14 years) | -0.370 | 0.206 | -.9453 - 0.205 |
| Quartile 3 (15-28 years) | -0.424 | 0.164 | -1.022 - 0.1745 |
| Quartile 4 (over 29 years) | -0.534 | 0.098 | -1.166 - 0.0990 |
| **Education** |  |  |  |
| Low (≤9 years) | -0.734 | 0.143 | -1.717 - 0.250 |
| Medium (10-12 years) | -0.103 | 0.663 | -.570 - 0.363 |
| High (>12 years) (reference) |  |  |  |
| Other | -0.443 | 0.052 | -2.899 - 0.0126 |
| **Alopecia areata only (vs alopecia totalis/iniversalis)** | -0.324 | 0.158 | -.776 - 0.127 |
| **Treatment satisfaction** |  |  |  |
| Dissatisfied | 1.182 | **<0.001** | 0.555 - 1.809 |
| Neutral (reference) |  |  |  |
| Satisfied | 1.739 | **0.004** | 0.554 - 2.924 |
| **Constant** | 7.829 | <0.001 | 6.769 – 8.485 |
| Number of observations | 245 |  |  |
| R-squared | 0.40 |  |  |

## Table SIV. Linear regression of factors influencing total cost of alopecia areata, Sweden (cost is in logarithm form)

| **Variables** | **β** | **p-value** | **95% CI** |
| --- | --- | --- | --- |
|  |  |  |  |
| **Male (vs female)** | 0.714 | 0.454 | -1.185 - 2.612 |
| **Age (years)** |  |  |  |
| 18-39 | -0.272 | 0.453 | -0.991 - 0.448 |
| 40-59 (reference) |  |  |  |
| 60- | 0.241 | 0.695 | -0.984 - 1.466 |
| **Years since diagnosis** |  |  |  |
| Quartile 1 (0-5 years) (reference) |  |  |  |
| Quartile 2 (6-14 years) | -0.467 | 0.288 | -1.340 - 0.406 |
| Quartile 3 (15-28 years) | 0.225 | 0.616 | -0.670 - 1.120 |
| Quartile 4 (over 29 years) | -1.038 | 0.051 | -2.083 - 0.007 |
| **Education** |  |  |  |
| Low (≤9 years) | -1.102 | 0.189 | -2.762 - 0.558 |
| Medium (10-12 years) | 0.175 | 0.632 | -0.553 - 0.902 |
| High (>12 years) (reference) |  |  |  |
| Other | 1.267 | 0.109 | -0.291 - 2.826 |
| **Alopecia areata only (vs alopecia totalis/universalis)** | 0.146 | 0.673 | -0.543 - 0.835 |
| **Treatment satisfaction** |  |  |  |
| Dissatisfied | 1.490 | **0.006** | 0.443 - 2.538 |
| Neutral (reference) |  |  |  |
| Satisfied | No values |  |  |
| **Constant** | 7.544 | <0.001 | 6.259 - 8.830 |
| Number of observations | 65 |  |  |
| R-squared | 0.27 |  |  |
